# The N-terminal region of photocleavable peptides that bind HLA-DR1 determines the kinetics of fragment release

**DOI:** 10.1371/journal.pone.0199704

**Published:** 2018-07-02

**Authors:** Maria Pia Negroni, Lawrence J. Stern

**Affiliations:** 1 Program in Biochemistry and Molecular Pharmacology, University of Massachusetts Medical School, Worcester, Massachusetts, United States of America; 2 Department of Pathology, University of Massachusetts Medical School, Worcester, Massachusetts, United States of America; National Chiao Tung University College of Biological Science and Technology, TAIWAN

## Abstract

Major Histocompatibility Complex class II (MHC-II) molecules bind peptides and present them to receptors on CD4^+^ T cells as part of the immune system’s surveillance of pathogens and malignancy. In the absence of peptide, MHC-II equilibrates between peptide-receptive and peptide-averse conformations. The conversion between these forms has been postulated to be important in regulating cellular antigen presentation but has been difficult to study. In order to generate the MHC-II molecule HLA-DR1 in the peptide-receptive form, we designed and tested a series of photocleavable peptides that included the UV-sensitive 3-amino-3-(2-nitrophenyl)-propionate amino acid analog. They were intended to bind tightly to the HLA-DR1 MHC molecule, but to generate low-affinity fragments after UV exposure that would be released to yield HLA-DR1 in the peptide-receptive conformation. We were able to identify photocleavable peptides that bound tightly to HLA-DR1 and generated the peptide-receptive conformation after UV exposure. However, slow release of photocleaved peptide fragments from the binding site limited the rate of binding of an incoming labeled peptide and complicated kinetic measurements of the individual steps of the overall peptide binding reaction. Modification of the N-terminal region of the photocleavable peptide to reduce MHC-II pocket or H-bonding interactions allowed for generation of the peptide receptive form immediately after UV exposure with peptide fragments neither retained within the site nor interfering with binding of an incoming peptide. However this was achieved only at the expense of a substantial reduction in overall peptide binding affinity, and these peptides had such weak interaction with HLA-DR1 that they were easily exchanged by incoming peptide without UV exposure. These results show that photocleavable peptides can be used to generate peptide-receptive HLA-DR1 and to facilitate peptide exchange in generation of specific peptide-MHC-II complexes, but that usage of these peptides for kinetic studies can be constrained by slow fragment release.

## Introduction

Major histocompatibility complex (MHC) molecules are membrane glycoproteins that bind short peptides and present them at the cell surface for interaction with receptors on T cells. This is part of the antigen presentation mechanism by which the immune system recognizes and clears pathogens and tumors. As T cells recognize peptide antigens only when bound to MHC proteins, studies of the MHC-peptide interaction are important for predicting and monitoring T cell-mediated immune responses. For more than a decade, extensive studies have been dedicated to understanding how peptides bind to MHC and how T cell receptors recognize the MHC-peptide complexes [1,2].

A major obstacle in the field is the instability of most MHC proteins in the absence of peptide. Consequently, folding in vitro in the absence of peptide is efficient for some particular MHC proteins [3,4], but for many MHC proteins appropriate in vitro folding conditions have not been established. Thus, in most cases MHC-peptide complexes have to be generated by exchange of pre-bound peptide or by de novo folding in the presence of peptide. For the exchange methods it often is difficult to find a peptide that binds sufficiently tightly to stabilize the MHC structure but sufficiently weakly so as to be exchanged easily. Moreover, even for MHC proteins that are stable in the absence of peptide, such as HLA-DRB1*01:01 (DR1) [5], the focus of this study, peptide binding reactions proceed slowly because of most of the preparation adopts a peptide-averse conformation [6–8]. To alleviate these problems, photocleavable MHC-binding peptides were developed [9,10]. These peptides incorporated the UV-sensitive β-amino acid 3-amino-3-(2-nitrophenyl)-propionic acid [11], or the α-amino acid(2-nitro)-phenylglycine [9,12], in place of a conventional α-amino acid. After photocleavage the peptide fragments bind more weakly to the MHC protein than the full-length peptide and can be easily exchanged for other peptides. This strategy allowed generation of many different MHC-peptide complexes for interrogation of T cells [10,13] and for study of molecular aspects of the MHC-peptide interaction [14,15].

**Fig 1:**
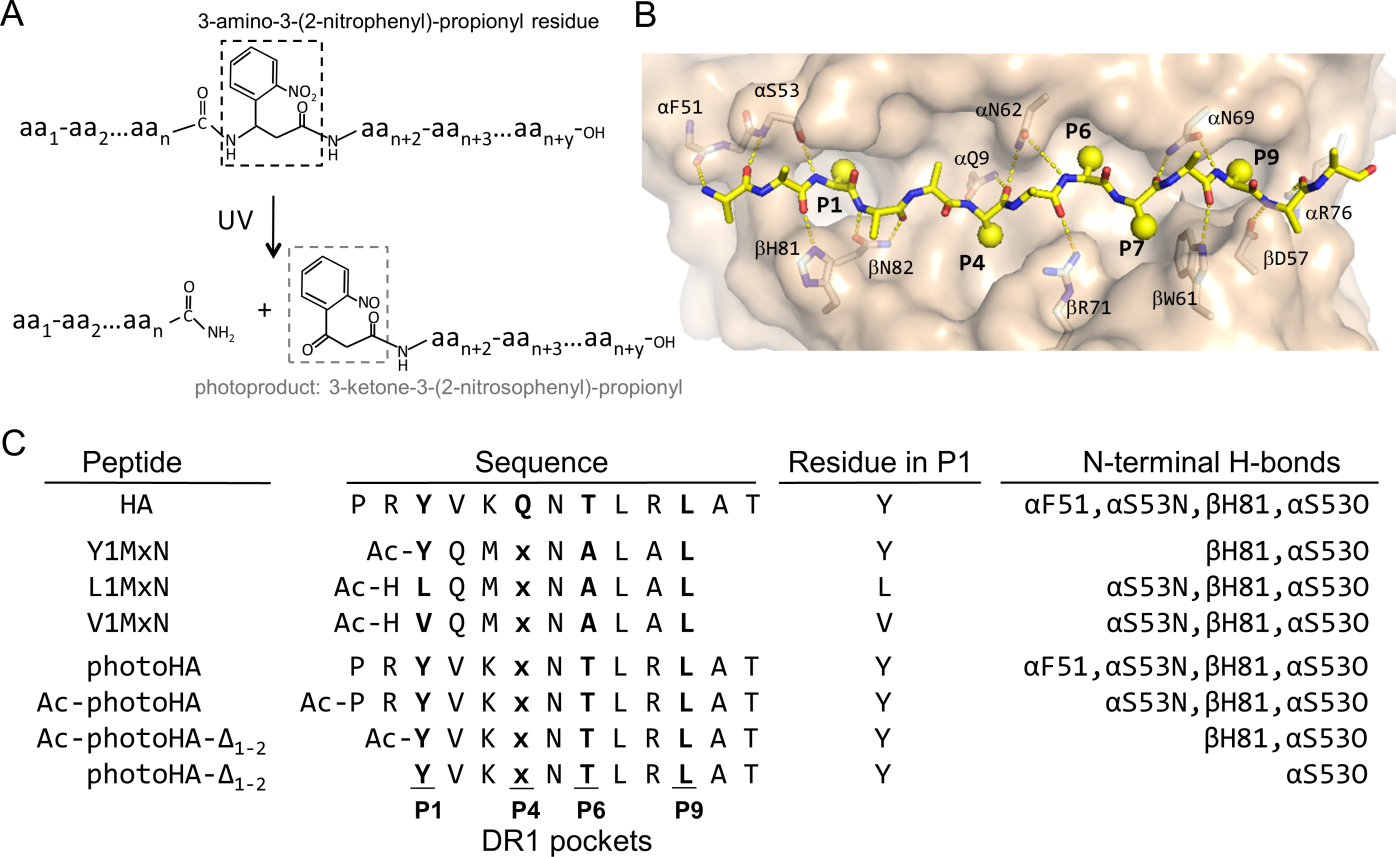
Photoclevable peptide design. (A) The photo-reactive group, 3-amino-3-(2-nitrophenyl)-propionyl (highlighted with a black dashed box) is incorporated as a residue in the peptide. When peptides with this group are exposed to UV light, the photo-reactive group is cleaved generating two peptide fragments. The N-terminal fragment will have an amide group and the C-terminal fragment will have a nitrosophenyl ketone (highlighted with a gray dashed box) on the site of the reaction. (B) DR1-peptide interactions. View of DR1 peptide binding groove, shown as a light-pink surface, occupied by a peptide shown as yellow sticks (blue: Nitrogen atoms, red: Oxygen atoms, yellow spheres: peptide side chains). DR1 peptide binding groove pockets are indicated as P1, P4, P6 and P9. The hydrogen bonds between the peptide backbone and conserved MHC II residues are shown as yellow dotted lines and DR1 residues involve in the hydrogen bonds are represented in sticks and labeled. (C) Peptides used along this study. Peptide nomenclature, sequences, residue occupying the P1 pocket and N-terminal hydrogen bonds between the peptide and DR1 are shown.

The photocleavable peptide strategy was first developed for MHC class I proteins (MHC I) and later generalized to MHC class II proteins (MHC II). MHC I proteins bind peptides of restricted length (usually 8–11 residues) and generally require both amino and carboxyl termini for stable binding [16–18]. Thus, a photocleavable group located at any site in the central region of a MHC I binding peptide should provide peptide fragments with low affinity after photoreaction. By contrast, MHC II proteins interact with peptide main chain and side chain groups all along the length of the peptide (Fig 1B) [19–21]. Moreover, short peptides can be bound efficiently provided that they occupy several of the peptide side-chain binding pockets [22,23]. To date, the photocleavable peptide strategy had been applied to MHC II protein only in a single case, using HLA-DRB1*15:01 (DR2b) and a peptide derived from human myelin basic protein to investigate the role of HLA-DM in peptide association [14].

We wanted to adapt the photocleavable peptide strategy for use with DR1, a common human MHC II protein that has been the focus of work on MHC II conformational changes and their relationship to peptide binding events [8,22,24–27]. DR1 has different binding constraints [28] than DR2b, previously used for development of photocleavable MHC-II binding peptides. The myelin basic protein sequence previously used has been shown to bind poorly to DR1, with IC50 ~ 700–20,000 nM depending on peptide length [29], despite binding tightly to DR2b (IC50 2–5 nM) [30–33]. Thus, we used a different peptide sequence optimized to bind to DR1 as a basis for development of a photocleavable peptide that could be used with DR1.

We intended to understand kinetic aspects of conformational changes that MHC II proteins undergo in the absence of peptide. So far, it is known that DR1 adopts peptide-receptive and peptide-averse conformations in reversible equilibrium [6,8,24]. Only the receptive form can bind peptide, limiting the overall rate of the MHC II peptide binding reaction. In order to perform studies on the kinetics of conversion between empty forms, we wanted to generate DR1 in the peptide-receptive, active conformation (DR1_a_) using DR1 loaded with a photocleavable peptide. Our strategy was to expose the DR1-photocleavable peptide complex to UV light, so the peptide is cleaved and the fragments are released from the peptide-binding grove, yielding empty DR1_a_. Two important caveats should be considered for this method to work as described. First, the fragments must have weak binding affinity so that they do not compete efficiently with incoming peptides. Second, the rate of peptide release has to be substantially faster than the MHC inactivation rate in order to build up a substantial concentration of the DR1_a_ form. Our initial attempts to design an effective photocleavable peptide resulted in fragments that were released very slowly from the peptide binding site. We found that weakening interactions between DR1 and peptide allowed faster fragment release but also allowed peptide exchange even without photocleavage. Thus, the photocleavable peptides we designed can be a good tool for investigation of DR1 peptide exchange, but fragment release kinetics must be taken into account and can limit the efficiency of generation of peptide-receptive DR1_a_.

## Results

### Photocleavable peptide design

In order to design a photocleavable peptide for DR1, we used the photolabile amino acid analog 3-amino-3-(2-nitrophenyl)-propionyl residue (Fig 1A) that under UV exposure rearranges so that an oxygen attacks the α-carbon cleaving the peptide backbone. This reaction yields an amide at the end of the N-terminal fragment and a nitrosophenyl ketone at the start of the C-terminal fragment (Fig 1A). We incorporated the 3-amino-3-(2-nitrophenyl)-propionyl group into a peptide designed to bind to DR1, taking into account the positions of DR1-peptide side chain interactions previously described (Fig 1B) [19,20,28]. MHC II proteins have four major peptide side-chain binding pockets, P1, P4, P6 and P9, named for the positions of the peptide side chains accommodated, counting from a large hydrophobic pocket (P1) that binds an aliphatic or aromatic side chain near the N-terminus of the peptide. In a study of the ability of peptides with different lengths to bind DR1 it was shown that peptides as short as four residues could still bind if they had side chains that interacted well with the P1 and P4 pockets [22]. Therefore, we decided to incorporate the photocleavable group at position P4, so that after UV exposure the N-terminal fragment ends at P3, which we expected would not have sufficient interaction with DR1 to remain bound. Other important MHC II-peptide interactions include hydrogen bonds between the peptide backbone and MHC II alpha and beta chain, which are distributed along the entire peptide length [21,34–37]. Some of these hydrogen bonds involve conserved glutamines and asparagines from the MHC II molecules that form 9 to 11 atoms rings and help holding the peptide in a polyproline type II helix [21]. The hydrogen bond formed between DR1 αQ9 and the amine and carboxyl group of the peptide residue at P4 (Fig 1B), forms a ring with 9 atoms. If a β-amino acid like 3-amino-3-(2-nitrophenyl)-propionyl residue were incorporated at the P4 position, the ring will be formed by 10 atoms, which most likely will still accommodate the polyproline type II helix of the peptide. With the photocleavable amino acid analog thus at the P4 position, we placed at the other pocket-interacting positions tyrosine at P1, alanine at P6, and leucine at P9. In each case the side chains of these residues are optimal for interaction with DR1[38]. At the intervening residues located at P2 (Gln), P3 (Met), P7 (Leu), and P8 (Ala), where peptide side chains make minor contacts with DR1 [20], we included residues with favorable side chains [38]without potentially reactive functional groups that might interfere with the desired photochemistry. At position 5, where the side chain does not interact with DR1, we included an asparagine group to increase solubility. We did not include any residues flanking the key MHC-interacting P1-P9 region, although we did include a N-acetyl group at the N-terminus to retain hydrogen bonds to the αS53 main chain and βH81 side chain. We refer to this peptide as Y1MxN (Fig 1C).

**Fig 2:**
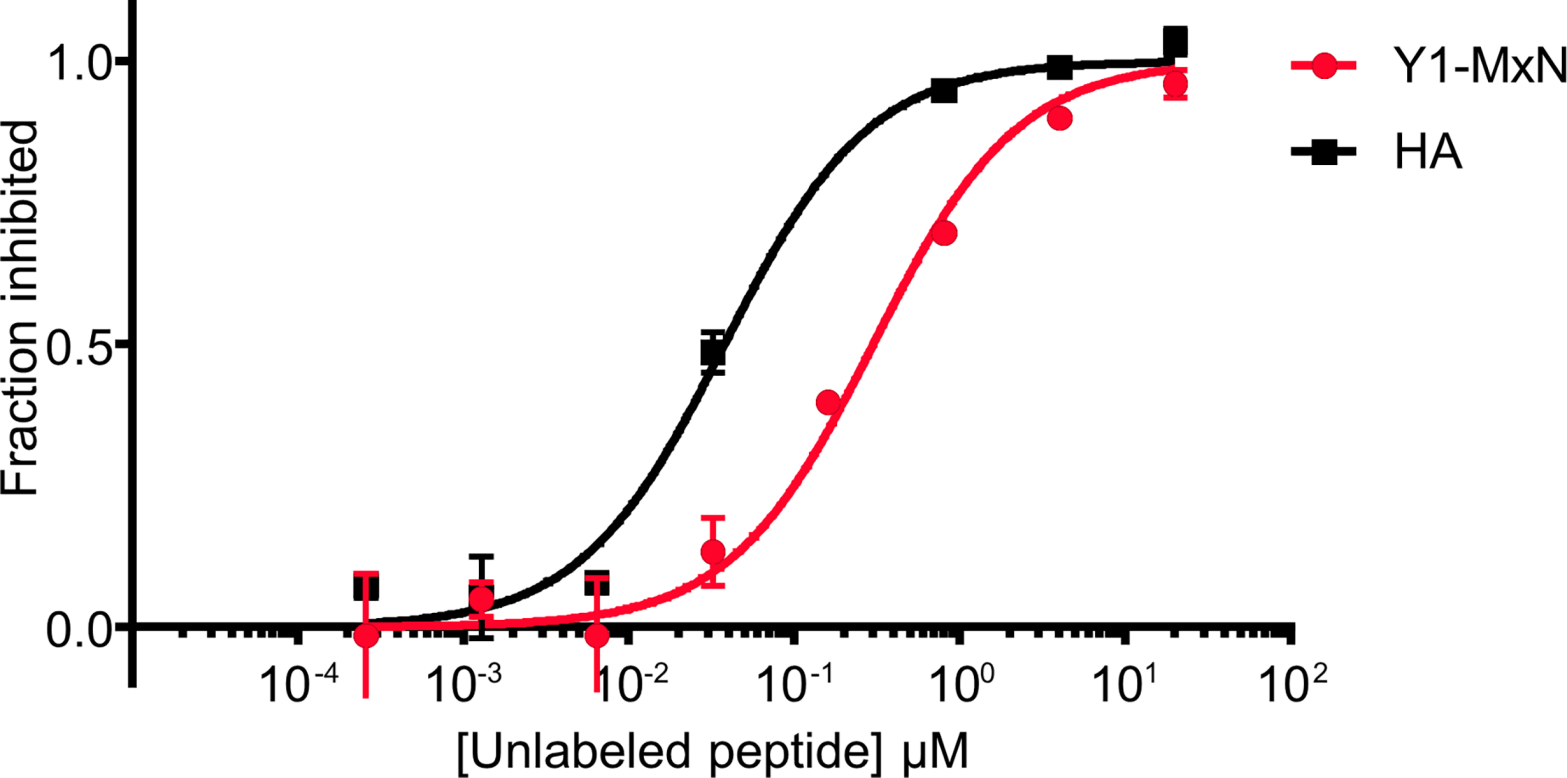
Y1MxN peptide can bind DR1. The ability of Y1MxN photocleavable peptide to bind DR1 was measured in a competition binding assay using a fluorescent high affinity binding peptide, HA (Ac-PRFVK*QNTLRLAT, where K* is lysine labeled with Alexa488). Different concentrations of Y1MxN or unlabeled HA were incubated for 3 days with a 100 nM DR1 and 25 nM Alexa488-labeled HA. Binding of the labeled peptide to DR1 was quantified by fluorescence polarization and the fraction of binding that was inhibited by the unlabeled peptide was calculated and plotted against unlabeled peptide concentration. Squares and circles show the mean and the error bars the standard deviation of two experiments done in triplicate.

We tested the ability of the Y1MxN peptide to bind to DR1 using a competition binding assay. DR1 was incubated with increasing concentrations of the photocleavable Y1MxN peptide and a constant concentration of a fluorescently-labeled non-photocleavable peptide (HA) based on a well-studied immunodominant HA_306-318_ viral epitope from influenza virus hemagglutinin, which binds tightly to DR1 with Kd estimated at 13 nM [39,40]. The fraction of fluorescent HA bound to DR1 in the presence of varying concentrations of Y1MxN was measured by fluorescence polarization assay [41](Fig 2). The Y1MxN peptide was able to compete with the labeled peptide for DR1 binding, but had reduced affinity, with an IC_50_ of 323 ± 23 nM as compared to 40 nM for HA (Table 1). This IC_50_ value, while higher than for the HA peptide, is in the range commonly observed for self- and foreign peptides bound to DR1 [42], and is similar to that observed for a photocleaveable myelin basic protein peptide binding to DR2b [14]. The reduced affinity did not interfere with the ability to isolate DR1-Y1MxN complex by gel filtration.

**Table 1:** Photocleavable peptides described in this study.

Peptide	Intact peptide ions		N-terminal ions		C-terminal ions		IC50 (nM)	Initial binding rate		
	Expected ^a^	Observed	Expected ^a^	Observed	Expected ^a^	Observed		No UV	5 min. UV	20 min. UV
Y1MxN	1157.63	-	482.17	-	676.45	-	323 ± 23	0.047±0.011	0.193±0.036	0.383±0.085
	1179.62 (MNa^+^)	1179.63	504.16 (MNa^+^)	504.17	698.44 (MNa^+^)	698.45				
L1MxN	1244.54	1244.58	569.25	569.23	676.45	-	4104 ± 298	0.523±0.043	1.920±0.431	1.676±0.279
V1MxN	1230.52	1230.57	555.24	555.27	676.45	-	5400 ± 341	0.513±0.050	0.531±0.051	0.668±0.077
photoHA	1623.87	1623.78	661.45	661.41	963.60	960.55 ^b^	92 ± 2	0.026±0.013	0.101±0.024	0.187±0.024
					979.59 (MH(O)^+^)	979.55				
Ac-photoHA	1666.01	1666.05	703.42	703.42	963.60	960.54 ^b^	134 ± 2	0.021±0.016	0.087±0.019	0.188±0.028
					979.59 (MH(O)^+^)	979.72				
Ac-photoHA-Δ_1–2_	1412.88	1412.76	450.27	450.24	963.60	960.46 ^b^	227 ± 23	0.043±0.023	0.136±0.039	0.391±0.056
					979.59 (MH(O)^+^)	979.51				
photoHA-Δ_1–2_	1370.85	1370.81	408.23	408.23	963.60	960.58 ^b^	1043 ± 88	0.340±0.011	0.473±0.021	1.240±0.100
					979.59 (MH(O)^+^)	979.48				

a. Expected masses are indicated for MH^+^ unless indicated

b. Unknown photoproduct with mass corresponding to MH^+^ minus 3 Da

To test whether the photocleavable Y1MxN peptide was cleaved to generate the expected fragments, we performed mass spectroscopy of the peptide before and after UV treatment. The mass spectra before UV exposure showed a major peak with an m/z corresponding to the intact peptide MNa^+^ ion. After UV exposure, MNa^+^ ions corresponding to the N-terminal or C-terminal expected fragments were detected (Table 1 and S1 Fig).

### Slow Y1MxN fragment release limits peptide binding to DR1

To test the ability of the photocleavable Y1MxN peptide to generate peptide-receptive DR1_a_, we compared the effect of UV treatment on DR1-Y1MxN, DR1 in complex with a non-photocleavable peptide HA, and peptide-free “DR1 empty”. For this purpose we used purified DR1-Y1MxN, DR1-HA and DR1 empty and exposed them to UV light at 4°C. The UV exposure was done using a long wavelength UV light (365 nm) to prevent potential protein damage from short-wavelength UV exposure. We kept the sample at 4°C during exposure to long-wavelength UV light, in order to stabilize the peptide-receptive DR1_a_ form. At elevated temperatures the conversion of DR1 to the peptide averse conformation is fast (τ = 8 min. at 37°C) but at 4°C the DR1_a_ can be stable for several days [7]. In this way, we can preserve the newly generated DR1_a_ in that conformation avoiding conversion to the peptide-averse form.

**Fig 3:**
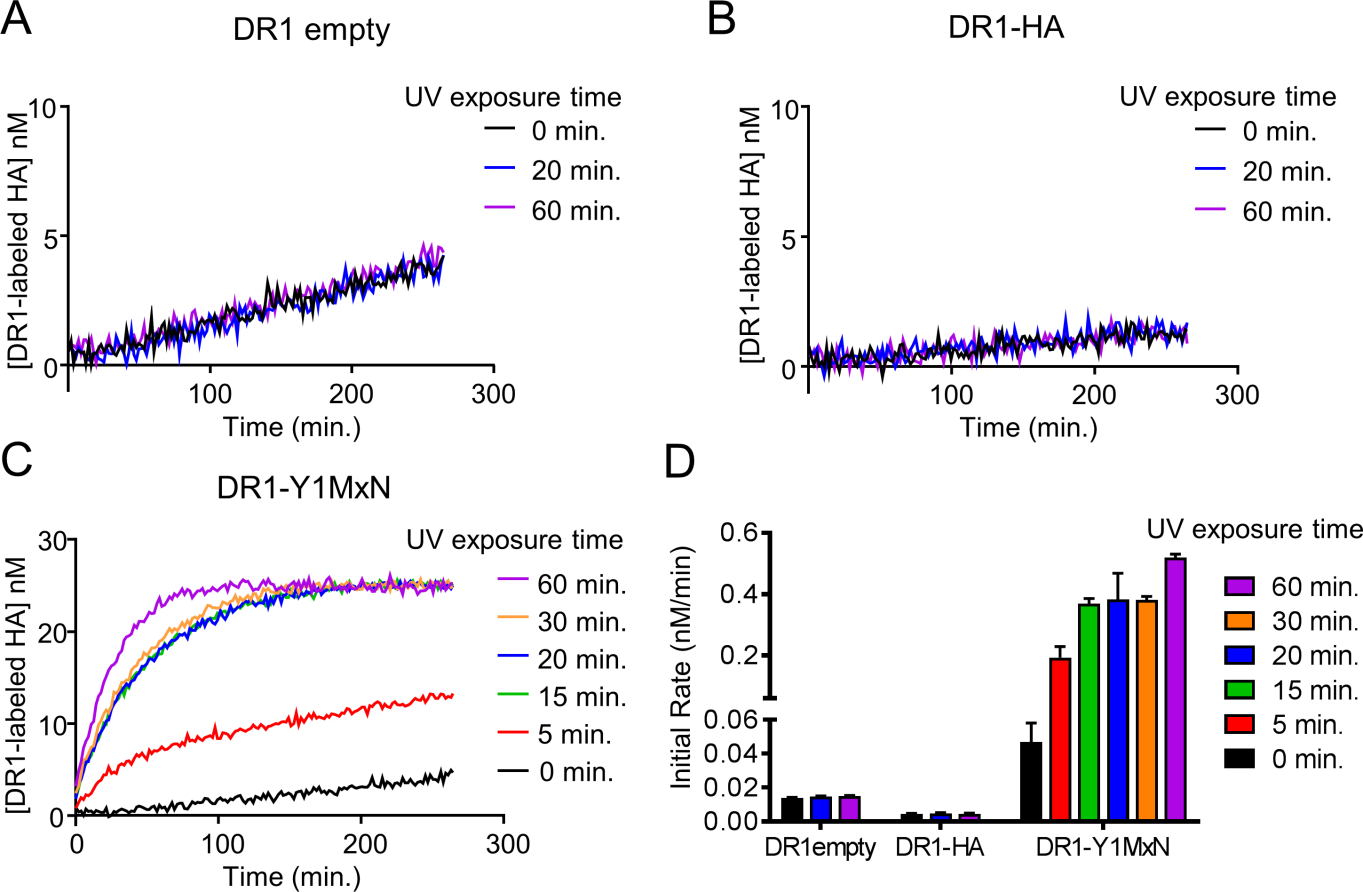
DR1-Y1MxN photocleavage generates DR1_a_. (A) 150 nM DR1 empty was exposed to UV light for different periods of time (20 minutes, blue trace or 60 minutes purple trace) or not exposed to UV (0 minutes, black trace), finishing all exposures simultaneously. After UV exposure, samples were mixed with 25 nM labeled HA and binding to DR1 was measured by fluorescence polarization. The concentration of labeled HA bound to DR1 is plotted vs. time. (B) Same as in panel A, but using DR1 bound to unlabeled HA. (C) DR1-Y1MxN was exposed to UV light for different periods of time (5 minutes: red trace, 15 minutes: green trace, 20 minutes: blue trace, 30 minutes: orange trace, and 60 minutes: purple trace) or not exposed to UV (black trace), finishing all exposures simultaneously. Then, samples were mixed with 25 nM labeled HA and binding to DR1 was measured by fluorescence polarization. The concentration of labeled HA bound to DR1 is plotted vs. time. Panels A-C show the mean of one representative experiment performed with duplicate or triplicate samples out of three independent experiments. (D) Initial peptide binding rates were calculated as the slope of a linear fit to the initial time points of the peptide binding data shown in panel A, B and C. Black bars show the values for DR1 empty, DR1-HA or DR1-Y1MxN binding to labeled HA without being exposed to UV light (0 min.). In red, green, blue, orange and purple bars are shown the binding rates of DR1 empty, DR1-HA or DR1-Y1MxN to labeled HA after being exposed 5, 15, 20, 30 or 60 minutes to UV light respectively. Bars represent the mean and the error bars the standard deviation of the initial rates calculated from three independent experiments done with triplicate samples.

After UV light exposure, we measured DR1 binding to fluorescently labeled HA at 37°C using fluorescence polarization assay. We observed that DR1 empty binds labeled HA at the same rate before (Fig 3A, black trace) and after 20 or 60 minutes of UV treatment (Fig 3A, blue and purple traces respectively) indicating that UV exposure does not modify the ability of DR1 to bind peptide. Similar results were shown for DR1-HA, for which binding to labeled HA behaves the same way for the sample not treated or treated 20 or 60 minutes with UV light (Fig 3B). We observed that DR1-HA binds labeled peptide slower than DR1 empty due to the slow off rate of the prebound HA. In contrast to DR1 empty or DR1-HA, DR1-Y1MxN bound peptide more efficiently after exposure to UV light (Fig 3C). After 5 min of exposure, DR1-Y1MxN exhibited increased binding of labeled HA (Fig 3C, red trace) as compared to a non-illuminated sample (Fig 3C, black trace), with additional illumination time (15 to 60 minutes) leading to increase rates of binding (Fig 3C). Initial rates of the peptide binding reaction after the different UV exposure times are shown in Fig 3D and Table 1. The observed increase in the initial rate of peptide binding after illumination indicates that after UV treatment, more DR1_a_ was present in the sample.

**Fig 4:**
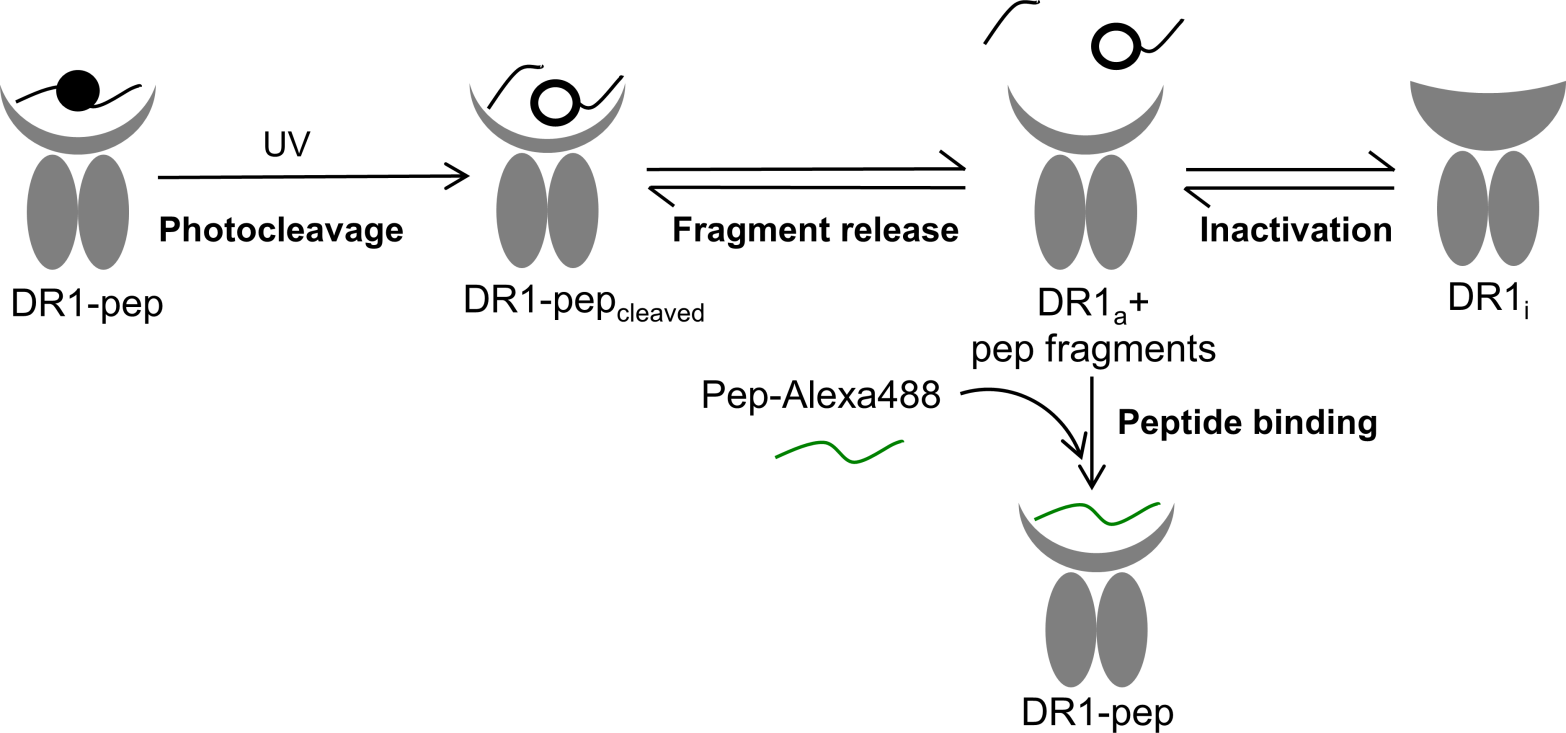
Generation of empty DR1 in its active conformation using a photocleavable peptide. DR1 is in its peptide receptive conformation when is bound to a peptide, in this case, a photocleavable peptide (DR1-pep). During UV exposure, photocleavage yields DR1 bound to two peptide fragments (DR1-pep_cleaved_). These fragments can get released from the peptide binding grove leaving DR1 in an active peptide receptive form (DR1_a_ + pep fragments) that can bind peptide. DR1_a_ can either inactivate, producing DR1 in its inactive peptide averse conformation (DR1_i_), or it can bind an incoming peptide, in this case a labeled peptide (Pep-Alexa488) used to track DR1-pep formation by fluorescence polarization.

Peptide photocleavage is expected to be a relatively fast reaction, and it was surprising that the maximum peptide-binding rate for DR1-Y1MxN was not achieved with 5 minutes of UV treatment. Five minutes of UV treatment yielded an intermediate rate (Fig 3C and 3D, red trace or bar), with the maximum rate achieved only with 15 minutes or longer UV treatment. In order to understand why 15 minutes or longer irradiation was needed to achieve the highest peptide binding rate, we considered which steps of the overall reaction pathway affect the accumulation of peptide-receptive, active DR1_a_ (Fig 4). The reaction starts with DR1 in complex with photocleavable peptide which holds the protein in the peptide-receptive conformation (DR1-pep Fig 4). During UV exposure, photocleavage yields DR1 bound to the low affinity peptide fragments (DR1-pep_cleaved_ Fig 4), which are released to generate empty DR1_a_ (DR1_a_+ pep fragments Fig 4). This form, which can bind labeled peptide, is in equilibrium with peptide-averse DR1 inactive form that cannot bind peptide (DR1_i_, Fig 4). Thus, accumulation of DR1_a_ could be affected by many steps, including photocleavage of the photocleavable peptide bound to DR1, release of the photocleavable peptide fragments from the peptide binding groove and the conversion of DR1_a_ into DR1_i_. We evaluated the role of these processes in generation of peptide-receptive DR1_a_ to understand why extended illumination was necessary for maximum binding rates.

**Fig 5:**
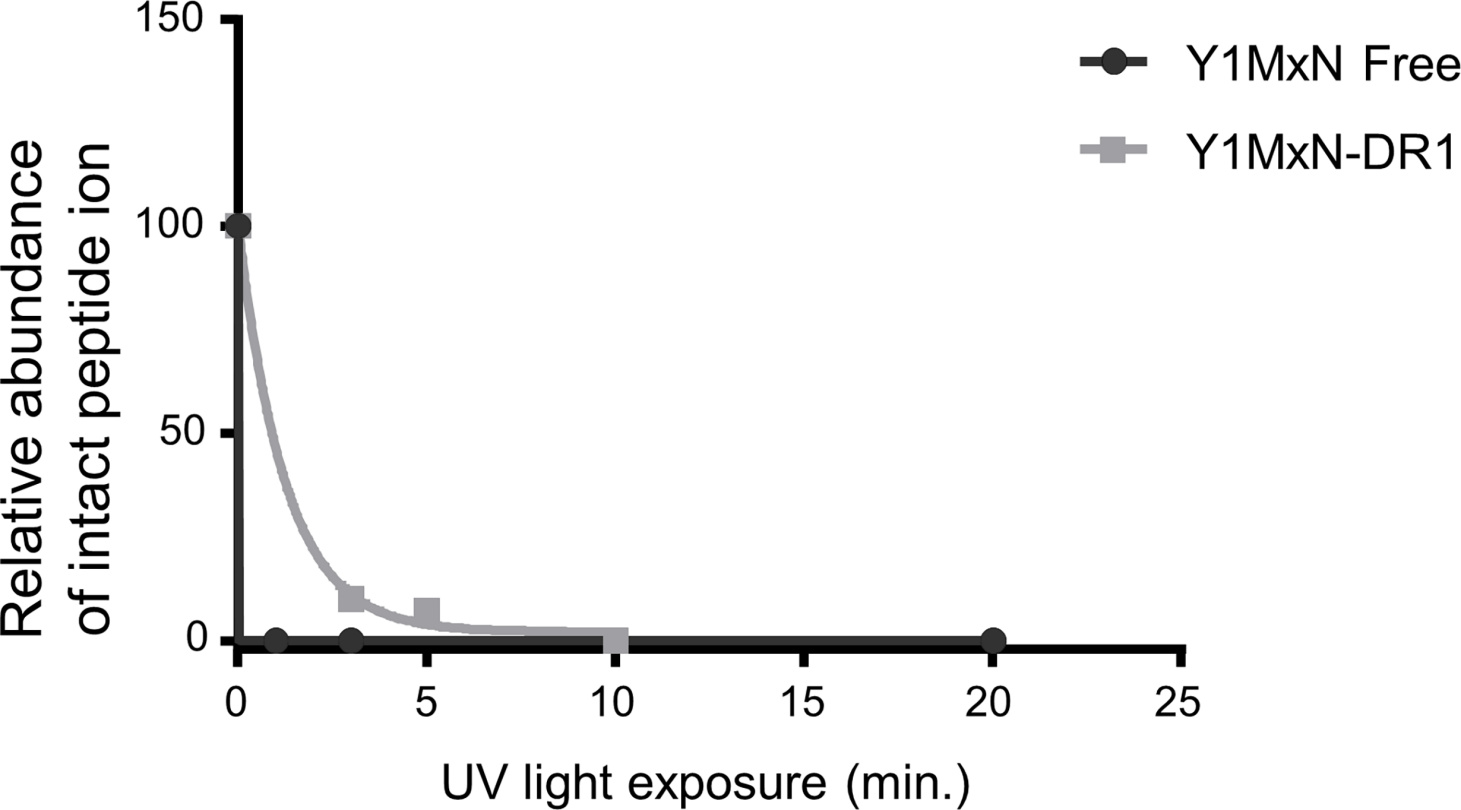
Y1MxN fragmentation occurs during short UV exposures. Y1MxN peptide free in solution (Y1MxN Free, black) or Y1MxN loaded on DR1 (Y1MxN–DR1, grey) were exposed to UV light for different periods of time and then each sample was analyzed by mass spectrometry using MALDI-TOFF. The relative abundance of the ion corresponding to the Y1MxN intact peptide present in each sample exposed 1, 3, 5, 10 and 20 minutes to UV light is plotted.

First we measured the rate of photocleavage of the Y1MxN peptide free in solution, or bound to DR1, to ensure that it was rapid as expected. We used mass spectrometry of Y1MxN free and bound samples before and after different lengths of UV exposure to measure the relative abundance of the ion that corresponds to the intact peptide (Fig 5). After 1 minute of UV exposure, the ion corresponding to the intact peptide was no longer detected in the Y1MxN free sample. For the Y1MxN loaded on DR1, photocleavage was slower than for the free peptide. After 3 minutes or 5 minutes of UV exposure, 90% and 93% of the peptide was cleaved, and after 10 minutes of UV treatment we could not detect ions corresponding to the intact peptide. This indicates that slow photocleavage of Y1MxN loaded on DR1 is not the reason why 15 minutes UV exposures was needed to achieve the maximum binding rate.

**Fig 6:**
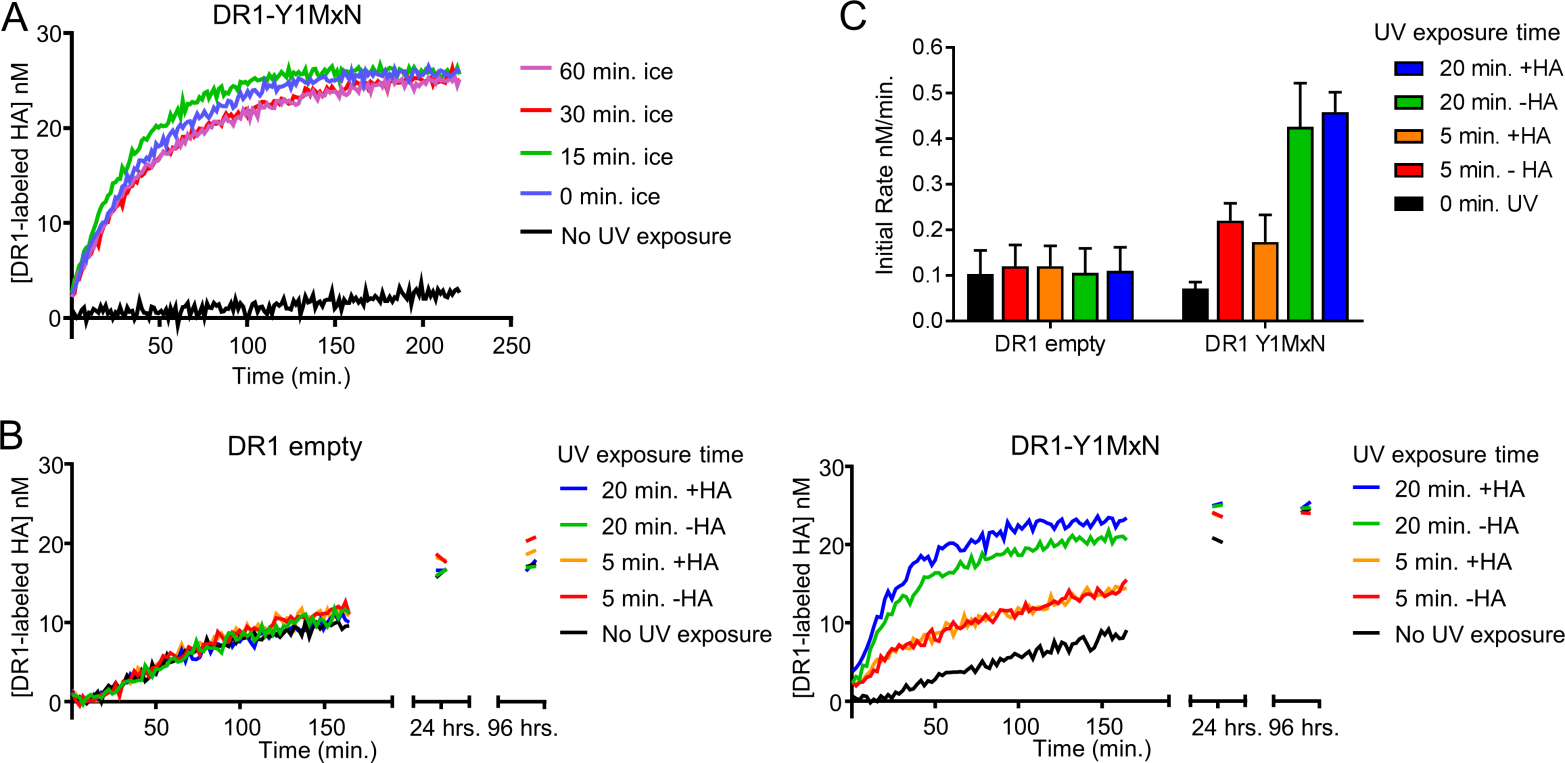
DR1_a_ generated after photocleavage of Y1MxN its stable at 4°C. (A) 150 nM DR1-Y1MxN was exposed to UV light on ice during 20 minutes and incubated on ice for different periods of time: not incubated (blue), 15 minutes (green), 30 minutes (red) and 60 minutes (pink). As a control, DR1-Y1MxN not exposed to UV light and kept always on ice was also tested (black trace). Then it was mixed with 25 nM labeled HA and binding to DR1 was measured by fluorescence polarization. The concentration of labeled HA bound to DR1 is plotted vs. time. Each data point shown in the plot is the mean of duplicate samples from one of two independent experiments. (B) 150 nM DR1 empty (left panel) or DR1-Y1MxN (right panel) were not exposed (black trace) or exposed to UV light for 5 (red and orange traces) or 20 minutes (green and blue traces) on ice. 25 nM labeled HA was either present during the UV exposure (“+HA” samples, orange and blue traces), or added after the exposure (“-HA” samples, red and green traces) and binding of labeled HA to DR1 was measured by fluorescence polarization. The concentration of labeled HA bound to DR1 is plotted versus time. Each data point shown in the plot is the mean of triplicate samples from one of three independent experiments. (C) The initial peptide binding rates were calculated as the slope of a linear fit to the initial time points of the peptide binding data shown in panel B and plotted as a bar graph. The bars represent the mean and the error bars the standard deviation of the initial rates calculated from three independent experiments done with triplicate samples.

Next, we addressed the inactivation of DR1_a_, to test if inactivation during the photocleavage reaction was limiting the generation of peptide-receptive DR1_a_. DR1 in complex with Y1MxN peptide was exposed to UV illumination for 20 minutes at 4°C, and then incubated for different periods of time at 4°C before addition of labeled peptide. We reasoned that if DR1_a_ were becoming inactivated at 4°C, we would observe a slower peptide binding rate after longer incubations at this temperature. In contrast we observed that the initial peptide binding rate of DR1 was not greatly affected by 15, 30 and 60 minutes of 4°C incubation after irradiation (Fig 6A, green, red and purple respectively) compared to no incubation (Fig 6A, blue trace). Thus, under these conditions, conversion to an inactive form does not occur appreciably even after 60 minutes of 4°C, consistent with a results from an earlier study using a different method of generation of peptide-receptive DR1_a_ [7]. As an additional test we performed the photocleavage step in the presence of labeled peptide, instead of adding labeled peptide after photocleavage as in previous experiments. We compared binding rates in these reactions to reactions where labeled peptide was added after photocleavage, reasoning that if DR1_a_ were becoming inactivated at 4°C, the presence of labeled peptide during irradiation would lead to additional labeled peptide binding. In contrast, we observed that binding rates were similar whether peptide was added during or after photocleavage (Fig 6B, compare -HA and +HA traces). Overall these results indicate that peptide-receptive DR1_a_ is stable at 4°C, and that inactivation during the photocleavage reaction is not likely to constrain generation of peptide-receptive DR1_a_.

Having measured photocleavage and the inactivation kinetics, and observing that photocleavage is almost completed at 5 minutes of UV exposure and that inactivation is not detected at 4°C, we suspected that the reason the peptide binding rate does not reach a maximum until 15 minutes or longer UV exposure was that peptide photofragments were retained in the DR1 peptide binding groove interfering with labeled HA binding. Because the major determinants of peptide binding to DR1 are side-chain binding pockets [19,22,37,43,44] and main-chain hydrogen bonding interactions [19,35–37,45] at the N-terminal side of the peptide binding groove, we suspected that retention of the N-terminal photoproduct was responsible. Thus we designed additional photocleavable peptides with reduced MHC-peptide interaction sites and tested these for ability to generate peptide-receptive DR1_a_.

### Y1MxN P1 pocket substitution

We attempted to promote fragment release by weakening the peptide interaction with the P1 pocket, which is the major determinant for peptide binding specificity for DR1 (and other HLA-DR1 allotypes) [22,34,44]. We replaced the tyrosine residue from the Y1MxN, expected to bind into the hydrophobic P1 pocket, for a leucine (L1MxN) or a valine (V1MxN). These substitutions are expected to lead to weaker MHC-peptide interaction, based on previously reported effects of substitutions at this position in other peptides [43,46]. We added a histidine on the N-terminal end of the peptide sequence to improve solubility and to allow an extra hydrogen bond with αS53N (Fig 1C). Both L1MxN and V1MxN exhibited the expected photocleavage after exposure to UV light, although C-terminal fragments were not detected (Table 1 and S1 Fig).

**Fig 7:**
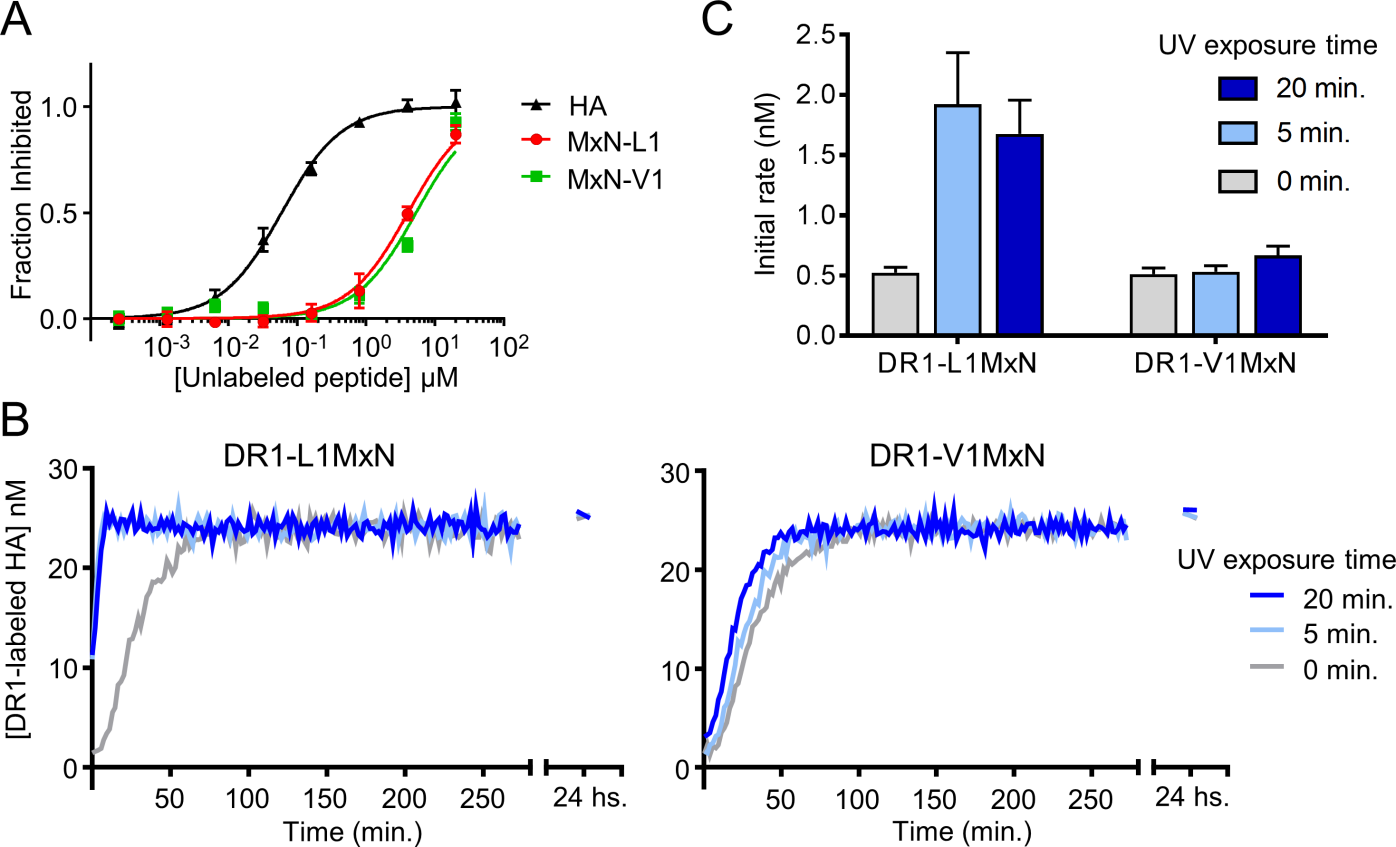
Photocleavable peptides with reduced interaction with DR1 P1 pocket generate DR1_a_ independently of UV exposure. (A) The ability of L1MxN and V1MxN peptides to bind DR1 was measured in a competition binding assay using labeled HA. Different concentrations of photocleavable peptides or unlabeled HA were incubated for 3 days with a 150 nM DR1 and 25 nM labeled HA. Binding of the labeled peptide to DR1 was quantified by fluorescence polarization and the fraction of binding that was inhibited by the unlabeled peptide was calculated and plotted against unlabeled peptide concentration. Squares, circles and triangles show the mean and the error bars represent the standard deviation of two independent experiments done with triplicate samples. (B) DR1-L1MxN (left panel) and DR1-V1MxN (right panel) were not exposed (grey trace) or exposed 5 (light blue) and 20 minutes (blue trace) to UV light on ice. Then, a 150 nM of each sample was mixed with 25 nM labeled HA and binding of the labeled HA to DR1 was quantified by fluorescence polarization. Concentration of the labeled peptide bound to DR1 is plotted against time. Each data point shown in the plot is the mean of duplicate samples from one of two independent experiments. (C) Initial peptide binding rates were calculated as the slope of a linear fit to the initial time points of the peptide binding data shown in panel B and plotted as a bar graph. The bars represent the mean and the error bars the standard deviation of the initial rates calculated from two independent experiments done with duplicate samples.

We evaluated the effect of the P1 substitutions in DR1 binding affinity using the competition binding assay. As expected, both L1MxN and V1MxN bound to DR1 more weakly than did Y1MxN, with IC_50_ values of 4100 ± 300 nM and 5400 ± 340 nM respectively (Fig 7A and Table 1). We used purified DR1-L1MxN and DR1-V1MxN complexes to test the ability of the newly designed peptides to generate DR1_a_. The DR1 complexes were exposed for 5 or 20 minutes to UV light, and binding to labeled HA was measured by fluorescence polarization (Fig 7B). After 5 minutes of UV light treatment (Fig 7B left panel, light blue trace), DR1-L1MxN showed a binding curve that behaves the same as the binding curve of the complex treated 20 minutes with UV light (Fig 7B left panel, dark blue trace). Similar results were observed for the V1MxN peptide, where labeled HA binding curves for the DR1-V1MxN exposed 5 or 20 minutes to UV light were similar to each other (Fig 7B right panel). In contrast to DR1-Y1MxN complex, which reached the maximum peptide binding rate with UV incubations of 15 minutes or longer (Fig 3C), L1MxN and V1MxN were able to induce the fastest peptide binding rate with 5 minutes of UV treatment (Fig 7C and Table 1). This indicates that these two peptides with weaker interaction with the DR1 P1 pocket are cleaved, released, and yield DR1_a_ within 5 minutes of UV treatment. However, we observed that DR1-L1MxN and DR1-V1MxN without UV treatment also had a fast peptide binding rate (Fig 7B, gray traces), indicating facile exchange of peptide even in the absence of photocleavage. The binding rate of DR1-L1MxN and DR1-V1MxN in the absence of irradiation (Fig 7C, grey bars) in fact was comparable to the binding rate of DR1-Y1MxN after 15 minutes of UV treatment (Fig 3D, green bar). This result shows that weakening the P1 pocket interaction in L1MxN and V1MxN peptides made them bind so weakly to DR1 that even without being photocleaved, they were easily released from the peptide binding groove and replaced by labeled HA. Thus, by replacing the peptide residue that interacts with the P1 pocket, we were able to reduce photocleaved peptide fragment retention, however this also had the unexpected and undesired effect of increases spontaneous peptide exchange.

### Reducing hydrogen bonds by N-terminal truncation

**Fig 8:**
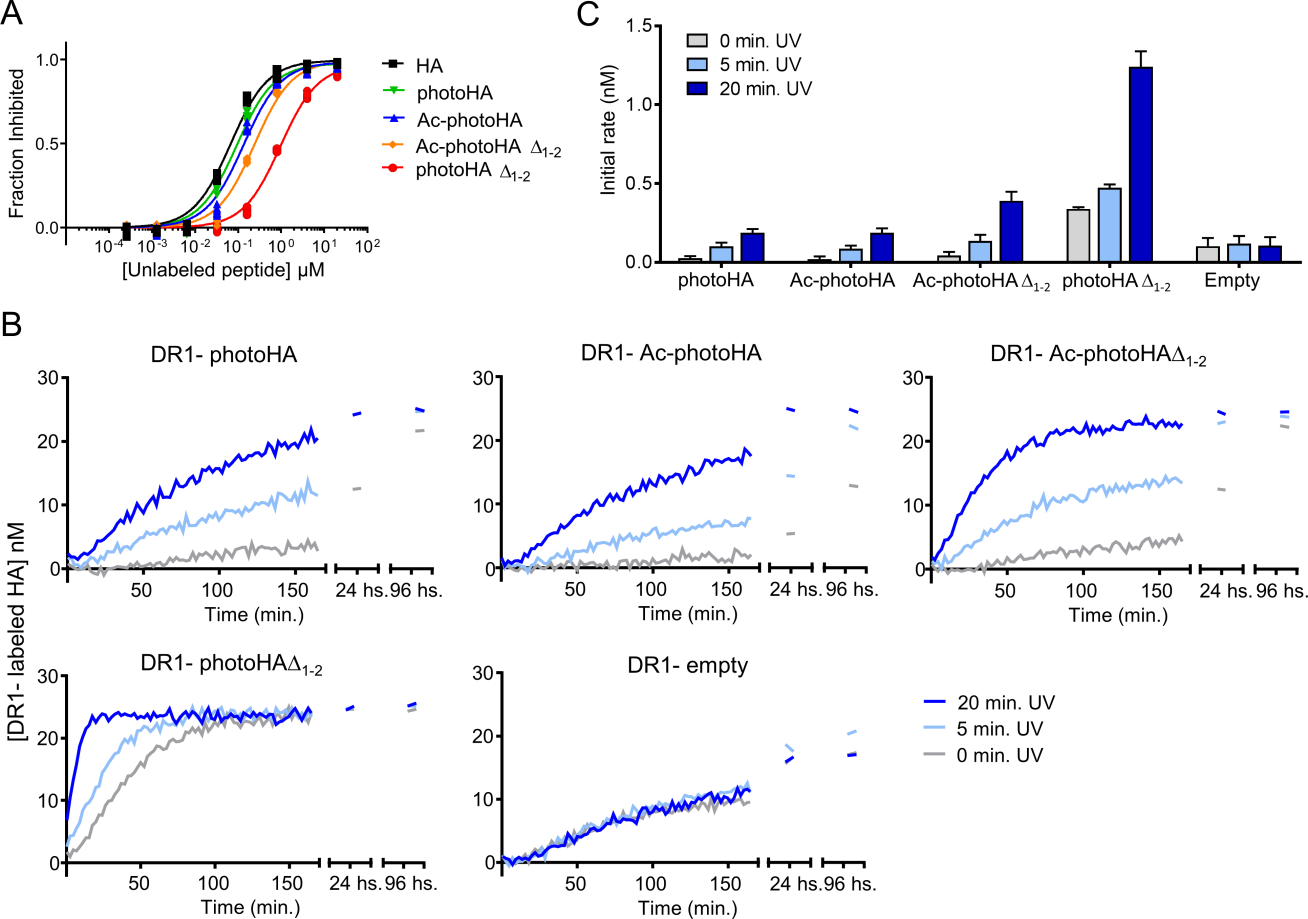
Photocleavable peptides with bigger N-terminal truncations generate DR1_a_ more efficiently but independent of UV exposure. (A) The ability of photoHA, Ac-photoHA, Ac-photoHAΔ_1–2_ and photoHAΔ_1–2_ to bind DR1 was measured in a competition binding assay using labeled HA. Different concentrations of photocleavable peptides or unlabeled HA were incubated for 3 days with a 150 nM DR1 and 25 nM labeled HA. Binding of the labeled peptide to DR1 was quantified by fluorescence polarization and the fraction of binding that was inhibited by the unlabeled peptide was calculated and plotted against unlabeled peptide concentration. Symbols show the mean and the error bars the standard deviation of three replicate samples from one representative experiment, out of two independent experiments. The mean IC_50_ from the two experiments is shown in Table 1. (B) DR1 loaded with photoHA, Ac-photoHA, Ac-photoHAΔ_1–2_, photoHAΔ_1–2_ or DR1 empty were not exposed (grey trace) or exposed 5 (light blue) and 20 minutes (blue trace) to UV light on ice. Then, a 150 nM of each sample was mixed with 25 nM labeled HA and binding of the labeled HA to DR1 was quantified by fluorescence polarization. Concentration of the labeled peptide bound to DR1 is plotted against time. Each data point shown in the plot is the mean of one representative experiment done with duplicate samples out of three independent experiments. (C) Initial peptide binding rates were calculated as the slope of a linear fit to the initial time points of the peptide binding data shown in panel B and plotted as a bar graph. The bars represent the mean and the error bars the standard deviation of the initial rates calculated from three independent experiments done with duplicate samples.

Since reducing the strength of the interaction between the peptide and the DR1 P1 pocket made the photocleavable peptide susceptible to exchange even in the absence of photocleavage, we explored modification of MHC-peptide hydrogen bonding in an effort to promote release of peptide photoproducts and facilitate UV-driven production of peptide receptive DR1. We designed four additional photocleavable peptides with different capacity to form main chain hydrogen bonds with MHC at the N-terminal side of the peptide binding site (Fig 1C). For these peptides we switched to HA as a framework, because pocket and H-bonding contributions have been investigated in detail for this peptide in several studies [45,46]. These four peptides maintained the same core sequence (position 1 to 9) but differ in that they have sequential truncations at the N-terminal end preceding the residue that binds in the P1 pocket. Usually, the first two peptide residues preceding the P1 pocket form three main-chain hydrogen bonds with DR1 alpha and beta chain residues (αF51, αS53 and βH81) (Fig 1B). The N-terminal modifications on the different photocleavable peptides abrogate the formation of some or all of these hydrogen bonds (Fig 1C). The peptide photoHA can form all three bonds, Ac-photoHA cannot form the first hydrogen bond with αF51, Ac-photoHAΔ_1–2_ cannot form the first two bonds with αF51 and αS53N, and photoHAΔ_1–2_ peptide cannot form any of the first three hydrogen bonds with αF51, αS53N and βH81. We measured the binding affinity of these peptides in the fluorescence polarization completion assay (Fig 8A). Binding affinity was decreased for the photocleavable peptides with reduced number of hydrogen bonds between their N-terminal end and DR1. The largest effect was observed with the photoHAΔ_1–2_ peptide that shows an IC_50_ of 1043 ± 88 nM, followed by Ac-photoHA’Δ_1–2_ with an IC_50_ of 227 ± 23 nM. This result indicates that the hydrogen bond between the peptide and βH81 plays an important role in stabilizing the DR1-peptide interaction consistent with previous studies that showed that peptides losing the hydrogen bond with βH81 had a bigger increase in their k_off_ than when losing bonds with αF51 and αS53 [35–37,45,46]. To test whether these peptides were cleaved to generate the expected fragments, we analyzed them by mass spectroscopy before and after UV treatment. After UV exposure, ions corresponding to the expected N-terminal fragments and related to the C-terminal fragments were detected (Table 1 and S2 Fig).

We evaluated the effects of photolabile peptide N-terminal truncation on the labeled peptide binding rate taking into consideration the particular MHC-peptide H-bonds disrupted by the truncations. All of the DR1 complexes with the N-terminal truncated photocleavable peptides showed increased binding of labeled HA after UV exposure (Fig 8B). In general, greater increases were observed for the shorter peptides, with initial rate of binding after both 5 and 20 minutes UV exposure increasing in the series Ac-photoHA < photoHA < Ac-photoHAΔ_1–2_ < photoHAΔ_1–2_ (Fig 8C and Table 1). Complexes with the two longest peptides, DR1-Ac-photoHA and DR1-photoHA, both showed small increases in labeled peptide binding rates after UV treatment. These peptides differ in that the Ac-photoHA cannot form the αF51 hydrogen bond. Larger increases in labeled peptide binding rate after 5 minutes or 20 minutes illumination were observed for Ac-photoHAΔ_1–2_, which in addition cannot form the αS53N hydrogen bond. Much larger increases in labeled peptide binding rates were observed for the photoHAΔ_1–2_ complex, which cannot form any of the αF51, αS53N, or βH81 hydrogen bonds. However, this complex exhibited substantial exchange of labeled peptide in the absence of illumination, similar to the complexes with V1MxN and L1MxN. Thus, N-terminal truncation of a photocleavable peptide can modulate retention of fragments after photocleavage, but as before optimum fragment release was linked to an increase in spontaneous peptide release.

## Discussion

We sought a photocleavable DR1-binding peptide that would generate peptide-receptive DR1_a_ after UV exposure. The first peptide that we designed and tested, Y1MxN, included the photocleavable amino acid analog 3-amino-3-(2-nitrophenyl)-propionic acid at the P4 position to optimally disrupt MHC-peptide interaction. This peptide bound well to DR1 with IC_50_ ~300 nM, and underwent photocleavage with <5 minutes of long-wavelength irradiation to generate the expected fragments. Peptide exchange after photocleavage was much faster than for either peptide-free “empty” DR1 (25-fold) or for DR1 preloaded with a non-photocleavable peptide (78-fold). Peptide exchange reactions after 15–60 minute UV exposures were complete within approximately ~100 min after photocleavage of DR1-Y1MxN, as compared to empty DR1 or preloaded DR1-HA for which complete exchange requires more than 24 hrs. Thus photocleaved Y1MxN generated DR1_a_ that was able to bind incoming labeled peptide with a faster rate than DR1 empty, which is in equilibrium between DR1_a_ and DR1_i_ forms. For many applications of photocleavable peptides such as high-throughput generation of different peptide-MHC complexes [9,10], Y1MxN would be ideal.

One application of the photocleavable peptides described here could be in generation of MHC-peptide tetramer staining reagents as previously done for MHC I [9,13]. Tetramers of MHC-peptide complexes can be used to study their recognition by antigen receptors on T cells (TCRs) [10]. One potential concern with this approach might be that UV irradiation might cause protein damage that could interfere with recognition by TCRs. An advantage of the 3-amino-3-(2-nitrophenyl)-photocleavable group used in this study is that it is cleaved by a relatively long wavelength (300–340 nm) irradiation [11]. It was shown previously that MHC-I tetramers made using long-wavelength UV irradiation, as used here, were able to stain T cells as well as conventional MHC-peptide tetramers [9] indicating that the UV treatment did not damage the MHC protein or interfere with its ability to present peptides to the TCRs.

We were interested in using photocleavable peptides to generate DR1 in a peptide-receptive form for studies of peptide binding kinetics. In initial experiments with Y1MxN, the rate of peptide binding depended on the length of time of UV exposure, despite the photocleavage reaction itself being essentially complete in <1 min. Further experimentation ruled out formation of non-receptive empty forms as a factor limiting peptide binding kinetics, and suggested that slow release of photogenerated peptide fragments might be responsible. To evaluate this possibility, we designed peptides with reduced MHC-peptide interaction. Substitution of the tyrosine in the key P1 pocket position by less-optimal side chains resulted in faster peptide binding after photocleavage as compared to Y1MxN, ~4.6-fold for L1MxN and ~1.8-fold for V1MxN, confirming retention of N-terminal fragments after photocleavage as a factor limiting production of peptide-receptive DR1. However, the P1 substituted peptides exhibited greatly increased rates of peptide binding in the absence of photocleavage (~36-fold for L1MxN and ~38-fold for V1MxN), limiting their use for controlled generation of peptide-receptive DR1. We designed a second series of peptides with N-terminal truncations instead of P1 substitutions, reasoning that fragment binding after photocleavage could be reduced by removal of key MHC-peptide hydrogen bonds, as an alternative to P1 pocket substitutions. Hydrogen bonds at the N-terminal side of the DR1 peptide binding site have been shown in many previous studies by several groups to be important determinants of DR1 binding affinity [35–37,45], and sequential removal of these interactions indeed resulted in faster binding of labeled peptide after photocleavage. However, spontaneous peptide exchange in the absence of photocleavage also increased with sequential removal of N-terminal hydrogen bonds, and as for the P1 substitutions, the maximal rate of peptide binding was observed only for a peptide that exhibited a high degree of spontaneous exchange. The best compromise for many applications would appear to be Ac-photoHAΔ_1–2_, which bound peptide 1.2-fold faster after photocleavage than did Y1MxN, with only 4.1-fold increase in spontaneous binding. However, the initial rate of peptide binding after photocleavage for this peptide was substantially slower than for other peptides, (ex., 2.9-fold slower than DR1-photoHAΔ_1–2_ and 3.7-fold slower than L1MxN), indicating that for Ac-photoHAΔ_1–2_, fragment retention still limited the kinetics of generation of peptide-receptive DR1.

Our observations on the effects of weakening P1 pocket and N-terminal hydrogen bonding interactions are consistent with previous studies. Work done to identify DR1-peptide motifs using a M13 phage library, showed a strong preference for big hydrophobic residues such as tyrosine, tryptophan or phenylalanine at P1 pocket [38]. Replacing the tyrosine for an alanine at the P1 pocket of viral HA peptide caused an increase in its IC_50_ value and impaired the ability of the peptide to induce DR1 conformational changes related to peptide binding [22]. Replacing the tryptophan for a leucine or valine at the P1 pocket of the transplantation antigen HLA-A2(104–117) peptide, increased the IC_50_ of the peptide and increases its susceptibility to HLA-DM-mediated editing, indicating that the replacement weakens the affinity of the peptide for DR1 [43]. Similarly to these previous findings, when we replaced the tyrosine of Y1MxN for a leucine in L1MxN, or a valine in V1MxN, we observed an increase in the IC_50_ and a faster peptide fragment release. Our observations of the effect of altering N-terminal hydrogen bonds also aligned with previous findings. Stratikos et al. used peptides based on the HA sequence and truncated the N-terminal end or modified the peptide sequence by adding methyl groups to interfere with different conserved hydrogen bonds between the peptide and DR1 [45]. They observed that loss of hydrogen bonds involving αF51 and αS53N (corresponding to the first and second hydrogen bonds shown in Fig 1B) caused only a small increase on the peptide IC_50_, but the loss of the hydrogen bond with βH81 (corresponding to the third hydrogen bond in Fig 1B) had a dramatic effect [45]. This hydrogen bond, between DR1 βH81 and the peptide carbonyl group of the residue preceding the P1, has been shown by several groups to be a key interaction between DR1 and the peptide. Mutation of βH81 to an asparagine, caused a significant decrease in the dissociation time of pre-bound peptides compared to wild type DR1, indicating that the loss of a single hydrogen bond has a significant impact on DR1-peptide affinity [35–37,46]. Consistent with these studies, we also observed that truncated photocleavable peptides that lost hydrogen bonds with αF51 and αS53N have a small increase in IC_50_ with somewhat faster release of their fragments, but dramatic effects on both IC_50_ and fragment release were observed when the βH81 hydrogen bond was removed in addition to loss of αF51 and αS53N hydrogen bonds. The photocleavable peptide with all the three hydrogen bonds removed was easily replaced by labeled HA even without photocleavage, indicating a substantial increase in its dissociation lifetime, along with 10.3-fold increase in IC_50_.

Although the effect of P1 pocket and H-bonding interactions were expected based on previous studies, an unanticipated complication of photopeptide design in this system was that substitutions needed for efficient fragment release after photocleavage also weakened the overall DR1-peptide binding interaction to the point where spontaneous peptide release was observed. It is possible that a search of additional photocleavable peptide variants might identify one with a more optimal balance of peptide binding affinity sufficiently strong to prevent release in the absence of photocleavage with N-terminal fragment affinity sufficiently weak to allow for fast peptide fragment release. All of the peptides we tested had the photocleavable group at the P4 position, and it is possible that a full-length peptide with the photocleavable group at the P1 position, could generate a 2 residue N-terminal fragment and a C-terminal fragment that will be similar to the photoHAΔ_1–2_ peptide which has a fast spontaneous release. If this were the case, the intact peptide will have all the key DR1 interactions and neither of the fragments will be retained after UV treatment. However, the photocleavable group has two features that may weaken peptide binding: it lacks a beta carbon, making it more rigid and potentially not able to adopt the right orientation for occupying the entire depth of the P1 pocket, and it has an additional methylene in the peptide main chain (β-amino acid), potentially altering the hydrogen bond network in the key region surrounding the P1 region [25].

In the antigen presentation field, photocleavable peptides were first used by the Schumacher group to generate MHC I that could easily be loaded with peptides of interest after UV exposure [9]. Several photosensitive peptides for binding to four different MHC I were designed and studied [10]. Many peptides containing the photolabile 3-amino-3-(2-nitrophenyl)-propionic acid residue efficiently exchanged peptide after UV exposure, but many did not. Bakker et al. observed that some of the MHC I-peptide complexes containing photolabile groups did not unfold after UV exposure in the absence of exchange peptide, which is surprising considering that MHC I is unstable without a peptide. This may indicate that some of the MHC I-binding peptides carrying photolabile groups generated fragments that were not released after photoreaction, providing stability to the MHC I molecule against unfolding. However this issue was not addressed during that work [10]. In general, photocleavable peptides have been used with MHC I molecules to promote peptide binding efficiency [9,10,15,47,48], but neither kinetics of photoproduct release, kinetics of exchange peptide binding, nor rates of spontaneous release were measured in these studies.

In the only previous report in which a photocleavable peptide was used with a MHC-II protein, the complex studied was DR2b (HLA-DRB1*15:01) with a peptide derived from human myelin basic protein (MBP_85-99_) with the photocleavable group replacing the residue at P4 position (ENPVVHFXKNIVTPR) [14]. It was shown that after 2 minutes of UV exposure the parent peptide was almost undetectable while the peptide fragments could be identified, and photogenerated DR2b bound peptide faster after 2 minutes than after 10 minutes of UV exposure. Thus in this case the peptide is cleaved efficiently and the fragments do not seem to interfere with peptide exchange. In contrast, we observed that for DR1 (HLA-DRB1*01:01), photocleaved peptide complexes bind peptide faster after 20 minutes than after 5 minutes of UV exposure, and release of photocleaved fragment appears to limit the kinetics of peptide exchange. There are several differences between the system used by Grotenberg et al., and the one described in this work that might help to explain the different behavior observed. First, the P1 pocket in the DR2b is not as deep as in DR1 due to substitution of Glyβ86 by Val [49], and the interaction between the valine from the MBP derived peptide and the DR2b P1 pocket is likely to be weaker than the tyrosine from the HA derived peptides and the DR1 P1 pocket. Second, the P5 to P10 residues of the MBP derived peptide bound to DR2b are positioned higher in the groove than the corresponding HA residues bound to DR1, with loss of a MHC-peptide hydrogen bond at the P5 residue due to substitution of Argβ71 by alanine. Thus both N-terminal and C-terminal photolysis fragments of the MBP peptide would be expected to bind more weakly to DR2b than do the fragments of the HA peptide to DR1.

Photocleavable peptides containing the 3-amino-3-(2-nitrophenyl)-propionyl residue are a powerful tool for generation of MHC proteins loaded with different peptides and for studying peptide sequence preferences of different MHC molecules. However, in the use of these reagents, we suggest that peptide exchange kinetics can be limited by release of photocleaved peptide products, even when such fragments are designed for minimal retention. We observed that binding of photocleaved peptide fragment did not interfere with the efficiency of peptide exchange, only the kinetics. Thus retention of peptide fragments would not be expected to interfere with use of photocleavable peptides to facilitate peptide loading, which has been the major application of these reagents to date.

## Materials and methods

### Equipment

The equipment used for this study was Gilson preparative HPLC system, Shimadzu Biotech Axima TOF^2^ (Shimadzu Instruments), reverse HPLC (Agilent) and Victor X5 Multilabel plate reader (PerkinElmer, Shelton, CT).

### Protein expression and purification

HLA-DR1 (DRA*01:01/DRB1*01:01) extracellular domain was expressed in *Drosophila* S2 cells and purified as previously described by Sloan VS, et al. [50]. For this purpose we used stable transformants of *Drosophila* S2 cells that express and secrete a soluble form of HLA-DR1, which were a gift from Dr. E. Mellins (Stanford University Medical College). These cells are cultured in SF900 II SFM media with penicillin-Steptomycin at 27°C and are induced with 1 mM CuSO4 when they are 8-10x10^6^ cells/ml. After 6–7 days of induction, the supernatant is collected, filtered and HLA-DR1 is purified using an LB3.1 immuno-affinity column.

### Mass spectrometry

Peptides Ac-YQMxNALAL, Ac-HLQMxNALAL, Ac-HVQMxNALAL, Ac-PRYVKxNTLRLAT, PRYVKxNTLRLAT, Ac-YVKxNTLRLAT and YVKxNTLRLAT (21^st^ Century Biochemicals, Marlboro, MA) where Ac represents a N-terminal acetyl group and x corresponds to the photocleavable group, were analyzed by mass spectrometry before and after they were exposed to UV light using matrix-assisted laser desorption/ionization time-of-flight (MALDI-TOFF). The peptides were deposited directly onto the MALDI sample target with 1–2 μl of acetonitrile:0.1% TFA (80:20) followed by addition of 0.5μl of matrix solution which consisted of 5 mg/ml of alpha cyano- 4-hydroxy cinnamic acid (recrystallized) in acetonitrile: 0.1% TFA (50:50). Samples were allowed to air dry prior to insertion into the mass spectrometer. Peptides were analyzed in positive ion Reflectron mode. All spectra were processed with Mascot Distiller (Matrix Sciences, Ltd.).

### Peptide labeling

The HA_306-318_-derived peptide Ac-PRYVKQNTLRLAT, where Ac represents N-terminal acetyl group, was labeled using the free amino group at position K5. For this purpose, 2 mgs of peptide were dissolved in 400 μl of 150 mM sodium bicarbonate pH 9.8 and mixed with 1 mg of Alexa488-tetrafluorophenyl ester (Molecular Probes). After one hour incubation at room temperature, labeled peptide was purified by reverse HPLC (Agilent) using a C18 column (Jupiter 300A 00G-4053-E0) and a gradient of acetonitrile in 0.02% trifluoracetic acid.

### Determination of relative binding affinity (IC_50_)

HA, Y1MxN, V1MxN, L1MxN, Ac-photoHA, photoHA, Ac-photoHAΔ_1–2_ and photoHAΔ_1–2_ peptides (Fig 1C) were diluted in binding buffer (100 mM sodium citrate, 50 mM sodium chloride, 0.1% octyl β-D-glucopyranoside, 5 mM ethylenediaminetetraacetic acid, 0.1% sodium azide, 0.1 μg/ml phenylmethanesulfonyl fluoride, 0.2 mM iodoacetic acid, 1 mM dithiothreitol). Serial dilutions of these peptides were prepared in binding buffer, as triplicates in wells of a black polypropylene 96 well plate (Bio-one, Grenier). Then DR1 and HA-Alexa488 were added to each well to final concentrations of 100 nM and 25 nM, respectively, in a final volume of 200 μl. The plate was sealed with aluminum sealing foil (USA scientific 2998–7100), incubated at 37°C for 72 h, and fluorescence polarization was measured using a Victor X5 Multilabel plate reader (PerkinElmer, Shelton, CT). The fractional inhibition was calculated as [1- ((mP_sample_—mP_free_) / (mP_no inibitor_—mP_free_))], where “mP_free_” is the mP value obtained from a sample were the only species present is labeled-HA (~30 mP), and “mP_no inhibitor_” is the mP value obtained from a sample were the competitor is not present (~350 mP).

### Determination of peptide association rate

DR1 was loaded with different photocleavable peptides by incubating purified DR1 with five times molar excess of photocleavable peptide at 37°C for 3 days in binding buffer (described above). DR1-peptide complexes or DR1 peptide-free as isolated from insect cells, was purified by gel filtration using Sephadex-200 column. The peak corresponding to the monomeric fraction was isolated and diluted in binding buffer to 300 nM, right before UV exposure begins. These samples were exposed to UV light for different periods of time in a pre-chilled low binding, polypropylene V-shape bottom 96 well plate (Bio-one, Grenier) on ice in a 4°C cold room with the long-wavelength UV lamp (Blak Ray B100AP/R, UVP), held 6 cm above of the plate. This lamp has emission spectrum that ranges from ~310 nm-410 nm with λmax of 365 nm and a 40 nm full-width at half maximum. After exposure the plate was covered and incubated for 3 min at 37°C. Then 25 μl of the samples were transferred to a black, polystyrene half well area 96 well plate (Corning) in duplicates or triplicates along with 25 μl/well of pre-warmed labeled-HA (50 nM) in binding buffer. The final volume per well was 50 μl with final concentrations of 150 nM of DR1 and 25 nM of labeled-HA in binding buffer. The plate was immediately read in a Victor X5 Multilabel plate reader (PerkinElmer, Shelton, CT) once every 50 seconds during 3 h. The concentration of HA-Alexa488 bound to DR1 was calculated as the fraction of peptide bound times the concentration of labeled peptide. The fraction of peptide bound was calculated as: [(mP_sample_—mP_100% free_) / (mP_100% bound_—mP_100% free_)], where “mP_100% free_” is the mP value obtained from a sample with no DR1 present to bind peptide (23 mP), and “mP100% bound” is the mP value for fully bound DR-HA obtained from a sample with 1 μM DR1 and 25 nM labeled-HA (342 mP).

In cases where labeled HA was present during UV exposure, 30 μl of 300 nM DR1 were mixed with 30 μl of 50 nM labeled HA in the pre-chilled low binding, polypropylene V-shape bottom 96 well plate (Bio-one, Grenier). After UV exposure, 50 μl were transferred to the black, polystyrene half well area plate, the plate was sealed and incubated at 37°C for 3 minutes. Then the plate was read in a Victor X5 Multilabel plate reader once every 50 seconds during 3 hours. The concentration of labeled HA bound to DR1 was calculated as described above.

### Statistical analysis

Competitive binding assays were performed in 2–3 independent experiments, with each sample in each experiment assayed in duplicate or triplicate, as indicated in the figure legends. We used all the individual values from the repeats of all the experiments to calculate the average and the standard deviation.

For the time-dependent peptide binding curves, the mean values shown correspond to the repeats obtained from one experiment. The time courses shown are representative of the result obtained in multiple experiments.

To calculate the average and standard deviation of the initial peptide binding rates from several experiments done with duplicate or triplicate samples, we plotted the peptide binding data as the mean and standard deviation of the repeats of each individual experiment. In that way we obtained a graph for each of the independent experiments. Using those plots, we calculated the initial peptide binding rate as the slope of a linear fit to the initial time points. The errors from those initial rates correspond to the fitting error. We report the average and standard deviations of the initial rates from 2–3 replicate experiments, as indicted in the figure legends.

## Supporting information

S1 Fig Photocleavable peptides with P1 substitutions get cleaved as expected. MALDI-TOFF was used to analyze **A-** Ac-YQMxNALAL, **B-** Ac-HLQMxNALAL, **C-** Ac-HVQMxNALAL. For all the peptides, the top plot shows the mass spectrum of the intact peptide before UV exposure (No UV) and the bottom plot shows the mass spectrum after 60 minutes of UV exposure (UV) performed at 4°C. The expected masses of the intact peptide, the N-terminal and C-terminal fragments are indicated at the top of each peptide panel. m/z of the main ions are indicated in each plot and the reference to what species they belong is stated next to them.
(PPTX)

S2 Fig Photocleavable peptides with N-terminal truncations get cleaved as expected. MALDI-TOFF was used to analyze **A-** Ac-PRYVKxNTLRLAT, **B-** PRYVKxNTLRLAT, **C-** Ac-YVKxNTLRLAT and **D-** YVKxNTLRLAT. For all the peptides, the top plot shows the mass spectrum of the intact peptide before UV exposure (No UV) and the bottom plot shows the mass spectrum after 60 minutes of UV exposure (UV) performed at 4°C. The expected masses of the intact peptide, the N-terminal and C-terminal fragments are indicated at the top of each peptide panel. m/z of the main ions are indicated in each plot and the reference to what species they belong is stated next to them.
(PPTX)

S1 Dataset Data used to make all the plots shown in each figure. (XLSX)
