# Supplementary material for: The N-terminal region of photocleavable peptides that bind HLA-DR1 determines the kinetics of fragment release
Source: PLoS One. 2018 Jul 2;13(7):e0199704. doi: 10.1371/journal.pone.0199704 (PMC6028098; doi:10.1371/journal.pone.0199704)
Supplement: S1 Fig — MALDI-TOFF was used to analyze A- Ac-YQMxNALAL, B- Ac-HLQMxNALAL, C- Ac-HVQMxNALAL. For all the peptides, the top plot shows the mass spectrum of the intact peptide before UV exposure (No UV) and the bottom plot shows the mass spectrum after 60 minutes of UV exposure (UV) performed at 4°C. The expected masses of the intact peptide, the N-terminal and C-terminal fragments are indicated at the top of each peptide panel. m/z of the main ions are indicated in each plot and the reference to what species they belong is stated next to them. (PPTX) [file pone.0199704.s001.pptx]

## Slide 1
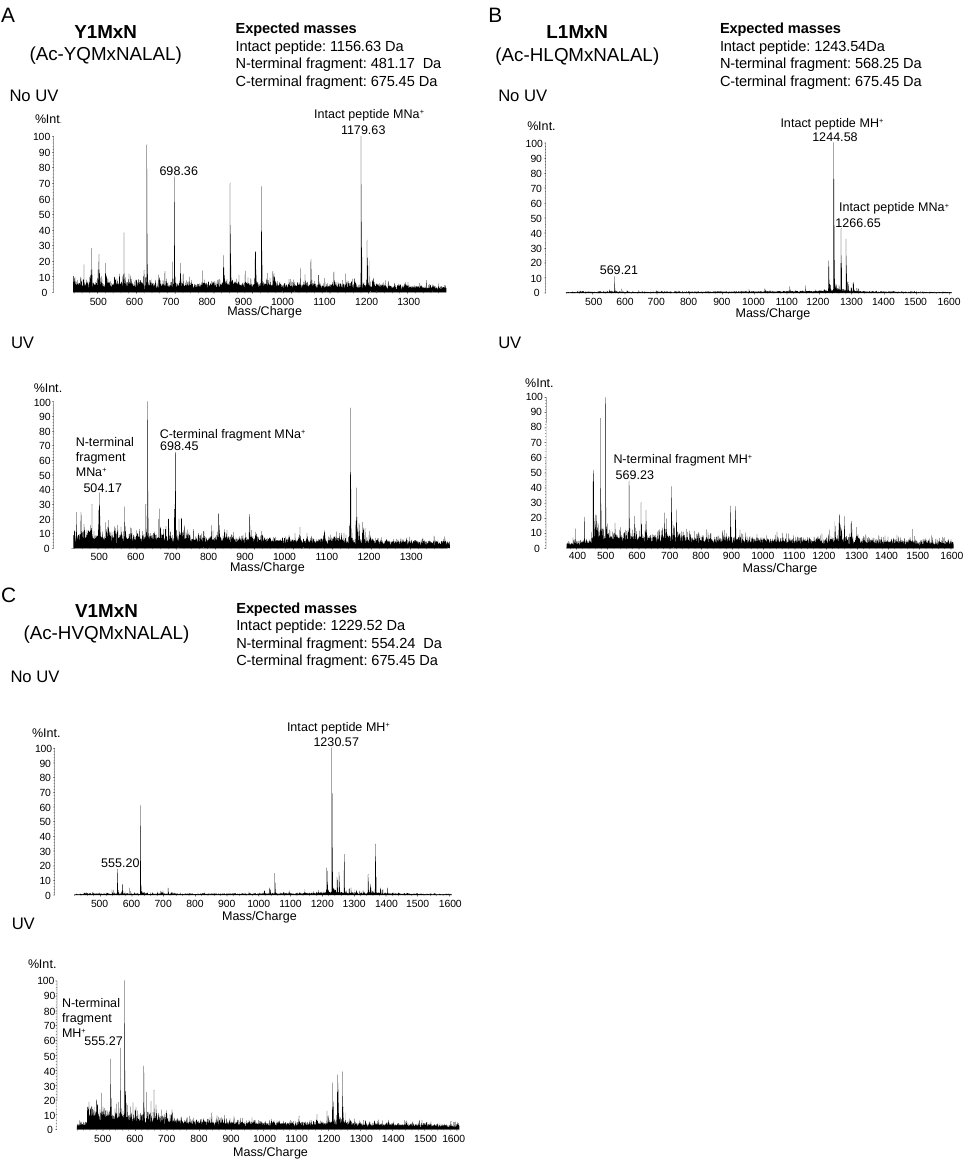

A
B
Y1MxN
(Ac-YQMxNALAL)
Expected masses
Intact peptide: 1156.63 Da
N-terminal fragment: 481.17 Da
C-terminal fragment: 675.45 Da
Expected masses
Intact peptide: 1243.54Da
N-terminal fragment: 568.25 Da
C-terminal fragment: 675.45 Da
L1MxN
(Ac-HLQMxNALAL)
No UV
No UV
Intact peptide MNa+
%Int.
1179.63
100
90
80
698.36
70
60
50
40
30
20
10
0
500
600
700
800
900
1000
1100
1200
1300
Mass/Charge
Intact peptide MH+
%Int.
1244.58
100
90
80
70
60
50
1266.65
40
30
20
569.21
10
0
900
800
500
600
700
1000
1100
1200
1300
1400
1500
1600
Mass/Charge
Intact peptide MNa+
UV
UV
%Int.
100
90
80
70
60
50
569.23
40
30
20
10
0
1000
400
500
600
700
800
900
1100
1200
1300
1400
1500
1600
Mass/Charge
%Int.
100
90
80
698.45
70
60
50
504.17
40
30
20
10
0
500
600
700
800
900
1000
1100
1200
1300
Mass/Charge
C-terminal fragment MNa+
N-terminal fragment MNa+
N-terminal fragment MH+
C
V1MxN
(Ac-HVQMxNALAL)
Expected masses
Intact peptide: 1229.52 Da
N-terminal fragment: 554.24 Da
C-terminal fragment: 675.45 Da
No UV
Intact peptide MH+
%Int.
1230.57
100
90
80
70
60
50
40
30
555.20
20
10
0
500
600
700
800
900
1000
1100
1200
1300
1400
1500
1600
Mass/Charge
UV
%Int.
100
90
80
70
555.27
60
50
40
30
20
10
0
500
600
700
800
900
1000
1100
1200
1300
1400
1500
1600
Mass/Charge
N-terminal fragment MH+
