# Supplementary material for: The N-terminal region of photocleavable peptides that bind HLA-DR1 determines the kinetics of fragment release
Source: PLoS One. 2018 Jul 2;13(7):e0199704. doi: 10.1371/journal.pone.0199704 (PMC6028098; doi:10.1371/journal.pone.0199704)
Supplement: S2 Fig — MALDI-TOFF was used to analyze A- Ac-PRYVKxNTLRLAT, B- PRYVKxNTLRLAT, C- Ac-YVKxNTLRLAT and D- YVKxNTLRLAT. For all the peptides, the top plot shows the mass spectrum of the intact peptide before UV exposure (No UV) and the bottom plot shows the mass spectrum after 60 minutes of UV exposure (UV) performed at 4°C. The expected masses of the intact peptide, the N-terminal and C-terminal fragments are indicated at the top of each peptide panel. m/z of the main ions are indicated in each plot and the reference to what species they belong is stated next to them. (PPTX) [file pone.0199704.s002.pptx]

## Slide 1
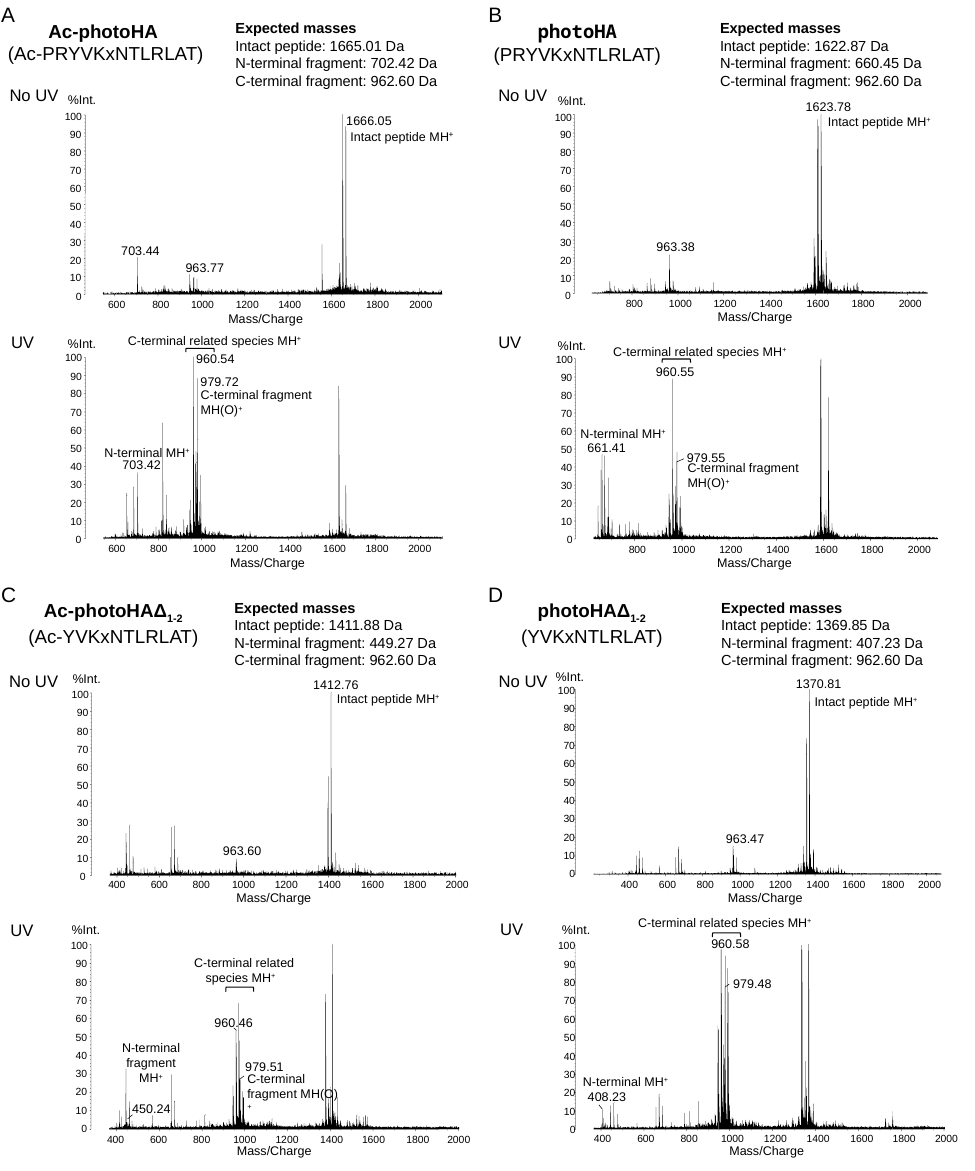

A
B
Ac-photoHA
(Ac-PRYVKxNTLRLAT)
Expected masses
Intact peptide: 1665.01 Da
N-terminal fragment: 702.42 Da
C-terminal fragment: 962.60 Da
Expected masses
Intact peptide: 1622.87 Da
N-terminal fragment: 660.45 Da
C-terminal fragment: 962.60 Da
photoHA
(PRYVKxNTLRLAT)
No UV
No UV
%Int.
1623.78
100
Intact peptide MH+
90
80
70
60
50
40
30
963.38
20
10
0
800
1000
1200
1400
1600
1800
2000
Mass/Charge
%Int.
100
1666.05
90
Intact peptide MH
+
80
70
60
50
40
30
703.44
20
963.77
10
0
600
800
1000
1200
1400
1600
1800
2000
Mass/Charge
C-terminal related species MH+
%Int.
100
960.54
90
979.72
80
70
60
50
N-terminal MH+
703.42
40
30
20
10
0
800
1000
1200
1400
1600
1800
2000
600
Mass/Charge
UV
UV
%Int.
C-terminal related species MH+
100
960.55
90
80
70
60
N-terminal MH+
661.41
50
979.55
40
30
20
10
0
800
1000
1200
1400
1600
1800
2000
Mass/Charge
C-terminal fragment MH(O)+
C-terminal fragment MH(O)+
C
D
Expected masses
Intact peptide: 1369.85 Da
N-terminal fragment: 407.23 Da
C-terminal fragment: 962.60 Da
Expected masses
Intact peptide: 1411.88 Da
N-terminal fragment: 449.27 Da
C-terminal fragment: 962.60 Da
Ac-photoHAΔ1-2
(Ac-YVKxNTLRLAT)
photoHAΔ1-2
(YVKxNTLRLAT)
No UV
No UV
%Int.
1370.81
100
Intact peptide MH+
90
80
70
60
50
40
30
20
963.47
10
0
400
600
800
1000
1200
1400
1600
1800
2000
Mass/Charge
%Int.
1412.76
100
Intact peptide MH+
90
80
70
60
50
40
30
20
963.60
10
0
400
600
800
1000
1200
1400
1600
1800
2000
Mass/Charge
C-terminal related species MH+
%Int.
960.58
100
90
979.48
80
70
60
50
40
30
N-terminal MH+
20
408.23
10
0
400
600
800
1000
1200
1400
1600
1800
2000
Mass/Charge
UV
UV
%Int.
100
C-terminal related species MH+
90
80
70
60
960.46
50
N-terminal fragment MH+
40
979.51
30
20
450.24
10
0
400
600
800
1000
1200
1400
1600
1800
2000
Mass/Charge
C-terminal fragment MH(O)+
